# Supplementary material for: Fabry-Perot Interferometer Based on a Fiber-Tip Fixed-Supported Bridge for Fast Glucose Concentration Measurement
Source: Biosensors (Basel). 2022 Jun 6;12(6):391. doi: 10.3390/bios12060391 (PMC9221283; doi:10.3390/bios12060391)
Supplement: Supplementary file 1 [file biosensors-12-00391-s001.zip › biosensors-1738238-supplementary.pdf]

biosensors MDPI

Supplementary Materials

Fabry-Perot Interferometer Based on a Fiber-Tip Fixed-Supported Bridge for Fast Glucose Concentration Measurement

Optimization of the bridge thickness

As shown in Figure S1, we performed stability tests on FP cavity structures with thicknesses of 3 μm, 5 μm, and 10 μm, respectively. The sensor was immersed in the solution and then rinsed with alcohol. Five minutes later, the spectra were stable and the data were recorded. We repeated the experiments four times for each sensor. The results showed that the spectra of sensors with a bridge of 3 μm and 5 μm shifted after the alcohol rinsing, while the spectra of the sensor with a bridge of 10 μm remain the same as the original wavelength after the alcohol rinsing. Thus, we confirmed that the 10 μm-thick micro-bridge did not deform after all the solution on it has evaporated.

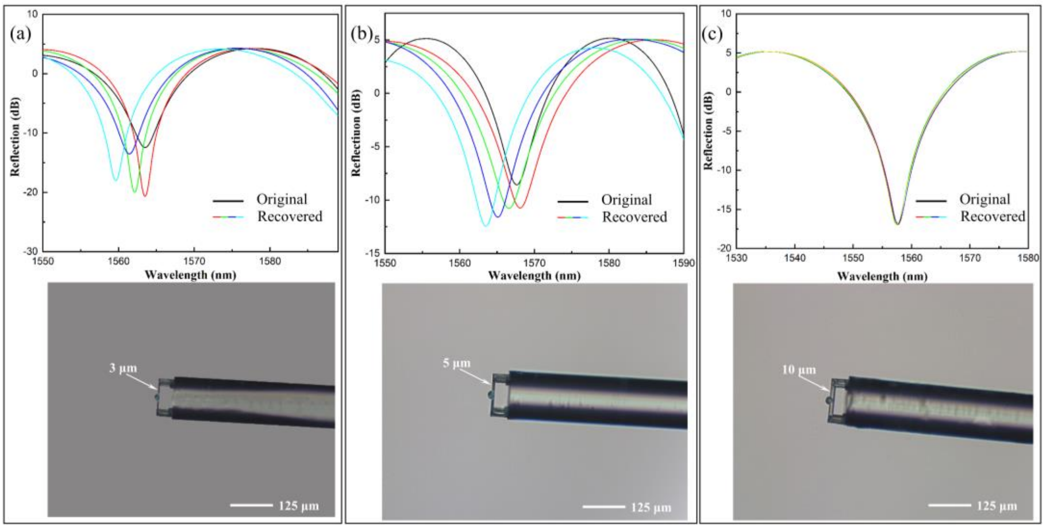

Figure S1. The comparison of the spectra and microscopy of the FP cavity structure with beam thickness of 3 μm (a), 5 μm (b), and 10μm (c). Scale bar: 125 μm.

Design of the bridge with hemisphere

In order to better demonstrate the effect of the central hemisphere, we have done a comparison experiment of the spectra of the fixed-supported bridge with and without the hemisphere, respectively, and the results are shown in Figure S2. It was obvious that the spectra from the FP cavity structure without hemisphere was thriple beam interference and there were a lot of small interference peak. However, the spectra from FP cavity structure with hemisphere was double beam interference, which was important for the performance of the sensor.

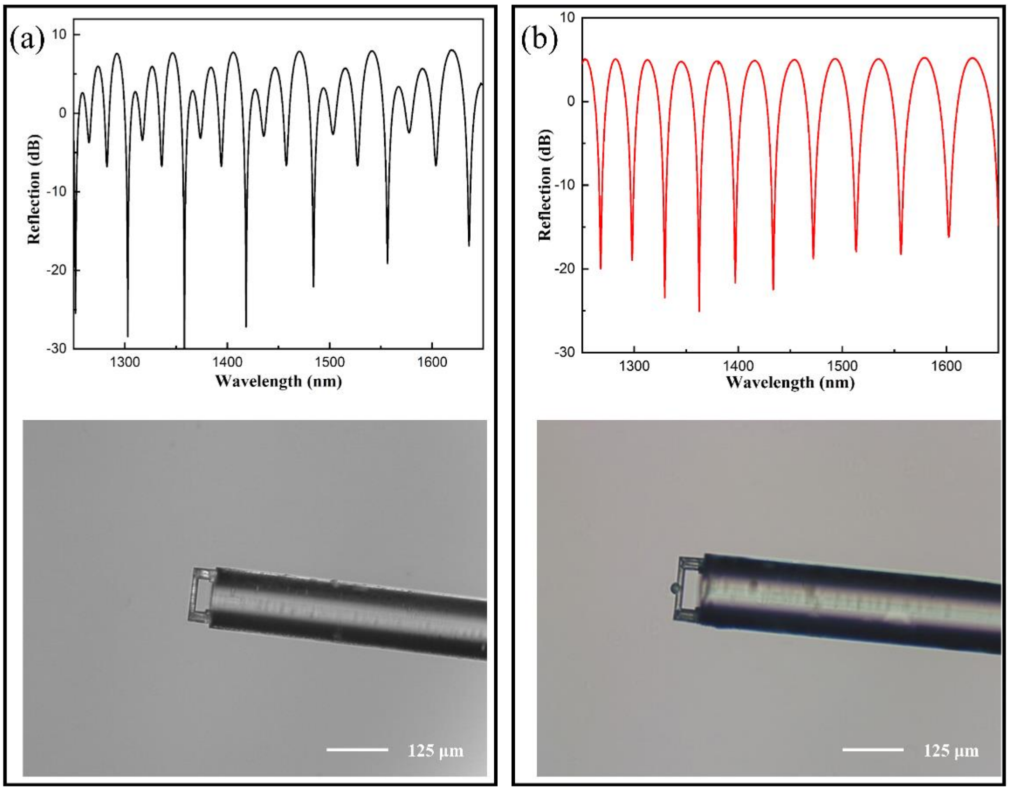

Figure S2. The comparison of the spectra and microscopy of the FP cavity structure without (a) and with hemisphere (b). Scale bar: 125 µm.
